# Local differential privacy protection for wearable device data

**DOI:** 10.1371/journal.pone.0272766

**Published:** 2022-08-17

**Authors:** Zhangbing Li, Baichuan Wang, Jinsheng Li, Yi Hua, Shaobo Zhang

**Affiliations:** School of Computer Science and Engineering, Hunan University of Science and Technology, Xiangtan, Hunan, People’s Republic of China; University College of Engineering Tindivanam, INDIA

## Abstract

Personal data collected by wearable devices contains rich privacy. It is important to realize the personal privacy protection for user data without affecting the data collection of wearable device services. In order to protect users’ personal privacy, a collection scheme based on local differential privacy is proposed for the collected single attribute numerical stream data. At first, the stream data points collected by the wearable device are censored to identify the salient points, and the adaptive Laplacian mechanism is used to add noise to these salient points according to the assigned privacy budget; then the collector reconstructs and fits the stream data curve to the noise-added salient points, so as to protect the personal privacy of the data. This scheme is experimented on the heart rate dataset, and the results show that when the privacy budget is 0.5 (i.e., at higher privacy protection strength), the mean relative error is 0.12, which is 57.78% lower than the scheme of Kim et al. With the satisfaction of user privacy protection, the usability of mean value estimation of wearable device stream data is improved.

## Introduction

Wearable devices are becoming more and more functional, providing people with convenient services while also recording and collecting various personal activity record data, including physiological data, activity data and environmental data. These data are available for individuals to monitor their own health and also contain a lot of personal and private information, such as personal heart rate, blood pressure, blood sugar, cholesterol, weight, personal activity range, habits, etc. Wearable device service providers may share the data with affiliated third-party companies in exchange for consistent service. Hackers may also directly obtain these data, so there is a risk of personal privacy leakage, such as exposure to health problems, living habits and scope of activities. The European Union’s General Data Protection Regulation (GDPR), implemented since May 2018, emphasizes the need for strong privacy guarantees for users when collecting and analyzing their usage data. The Personal Information Protection Law of the People’s Republic of China, implemented from November 1, 2021, also emphasizes that the collection of personal information should be limited to the minimum extent necessary to achieve the purpose of processing and regulates the collection of personal information. Therefore, the privacy protection of wearable device data collection has received increasingly serious attention in academic fields [1]. It is of great significance to realize the personal privacy protection for user data without affecting the data collection of wearable device services.

Wearable devices collect numerical data at a certain collection period, which grows over time to form a temporal stream dataset. Papageorgiou et al. [2] 2018 investigation results show that most apps do not strictly follow the law and they will send collected user datasets to partner third parties. To protect privacy, users do not want their real data to leave their devices. Local differential privacy techniques provide a solution to this problem by adding noise to the real data on the user side and then sending the noised data to the server side for aggregation. The main perturbation mechanism of local differential privacy is currently the random response mechanism [3], and there have been many research studies on local differential privacy, mainly applied in frequency estimation and mean value estimation. Erlingsson et al. (2014) [4] of Google proposed the RAPPOR mechanism, Bassily et al. (2015) [5] proposed the S-Hist algorithm for frequency estimation of categorical data. Duchi et al. (2013) [6, 7] proposed the MeanEst method for mean value estimation of numerical data; Nguyên et al. (2016) [8] proposed the Harmony algorithm for processing both categorical and numerical data.

Data collection for wearable devices belongs to the domain of mean value estimation for temporal stream data. Using local differential privacy techniques, each stream data is assigned a privacy budget to add noise protection, which leads to bad data usability. Meanwhile, with the increasing stream data, the privacy budget assigned to each stream data is getting smaller so that data usability is getting worse. Kellaris et al. (2014) [9] proposed w-event privacy mechanism to solve this issue, but there was a waste of privacy budget, which made the data usability decrease and affected the overall accuracy. Errounda et al. (2021) [10] applied w-event privacy mechanism on location statistics, but with the same unnecessary utility loss. Fang et al. (2020) [11] also based on this mechanism and proposed Local Differential Privacy Data Streaming Protocol (LDPS), adaptively invoked method to handle categorical and numerical data. However, the resource consumption is too large and not suitable for wearable devices. Wang et al. (2016) [12] demonstrated that the random response mechanism outperforms the Laplace mechanism in terms of mean square error. Kim et al. (2018) [13] applied local differential privacy to one-dimensional attribute heart rate data collection and used the Laplace mechanism to add noise to salient point data, but the data error was large and usability was not high. There will be a crucial issue on how to improve the data usability while protecting privacy.

In a nutshell, existing studies on local differential privacy protection for stream data are relatively few and inadequate. It will be the main point of this paper to study how to combine the ability of Laplace mechanism for stream data processing with the randomness of random response to improve the usability of data.

In this paper, we propose an improved scheme based on local differential privacy for personal health data collection, which solves the privacy protection problem of stream data collection on a single attribute. The scheme combines the Laplace mechanism and the random response mechanism, which makes the data more usable while protecting privacy. The main contributions of this paper are as follows:

1. We improve the approach of identifying salient points for stream data and obtain a data set that is closer to the original stream data curve.
2. In order to solve the problem of large noise that impacts data usability, we modify the noise addition mechanism by bringing in self-adaptive random values.
3. We improve the data reconstruction methods, which reduce the reconstruction data errors and increases the data usability.

The remainder of this paper starts with the Related work. The proposed approach is described in the section Wearable device data protection based on local differential privacy, and the experiments are done in the section Experiment and analysis. Finally, we close this paper with the Conclusion section.

## Related work

As technology evolves, data security is facing more and more attacks [14, 15]. The current data privacy protection technologies for wearable devices are mainly based on cryptography and perturbation [16].

The cryptography-based schemes are mainly homomorphic encryption and secure multi-party computation. These kinds of methods have high security but low efficiency. Wang et al. (2015) [17] used a medical system as an example to constructed a network model, a trust model and a security model for wearable sensor devices. She proposed a homomorphic encrypted data aggregation technique based on data segmentation technique which has high security but slightly high communication load. It is not suitable for wearable devices. Xu et al. (2020) [18] proposed a data security and privacy protection scheme based on the sanitizable signatures technology for the security and privacy protection of medical data in smart mobile medical scenarios. Wei et al. (2020) [19] proposed a group blind signature scheme in smart grid to accomplish conditional anonymity. Homomorphic encryption (HE) was used to verify the integrity of power data, which reduced the communication overhead between the control center and the smart meter, so as to protected the user privacy and consumption data of the smart grid. Wei et al. (2021) [20] summarized the security and privacy requirements for the security authentication of the intelligent terminal devices, proposed a privacy-preserving implicit authentication framework based on cosine similarity and partial homomorphic public key encryption, which utilized artificial intelligence methodology to sense the users’ behavior features from the mobile intelligent terminal. Liu et al. (2022) [21] proposed a novel privacy-preserving Dynamic Searchable Symmetric Encryption (DSSE) scheme for Intelligent IoT Healthcare (IIoTH) system. They constructed a secure index based on hash chain and realized trapdoor updates for resisting file injection attacks. They realized fine-grained search over encrypted personal health record (PHR) files database of attribute-value type, which made user searching the dynamic healthcare information from IIoTH system to protect the privacy. Hua et al. (2018) [22] applied secure multi-party computation to medical data generated by wearable devices to achieve fast and secure two-party query computation, which in turn forms an efficient privacy-preserving scheme for medical pre-diagnosis, but it also requires a large amount of computing resources.

The perturbation-based schemes are mainly anonymization techniques, centered differential privacy, and local differential privacy. These schemes have high computational efficiency, but the data accuracy may be biased. Anonymization techniques mainly provide protection for data by utilizing the generalization idea [23], where k-anonymity, l-diversity, and t-closeness are the main models. For example, Liu et al. (2018) [24] proposed a clustering-based k-anonymization method, which assigns similar records to the same equivalence set, making it more difficult to distinguish between records, and thus data accuracy will be biased. Zhou et al. (2022) [25] employed a block design technique to obfuscate various health indicators from the hospitals and the smart wearable devices, and introduced human-in-the-loop (HitL) to enable a privacy access of the health reports from the smart healthcare platform. Centered differential privacy is a scheme based on trusted third parties with two implementation mechanisms, namely Laplace mechanism for numerical data and exponential mechanism for non-numerical one [26]. For example, Yang et al. (2020) [27] proposed a privacy-preserving framework for student health data on smart wearable devices, improving the centered differential privacy technique by adding noise with a Laplacian mechanism and filtering the appropriate data for publication through shielding conditions. It reduces the possibility of an attacker finding abnormal data so as to infer the user’s privacy information. For that purpose, part of the data is lost, which affects the overall accuracy.

Local differential privacy is a scheme built on the user side, which mainly uses random response mechanism to protect the privacy of the data [3]. The current research field is related to statistical databases and divided into single-valued frequency estimation, multi-valued frequency estimation and mean value estimation. Erlingsson et al. of Google [4] proposed the RAPPOR mechanism, based on random response with Bloom filter, to count single-valued frequencies with high data usability but high computational cost. Li et al. (2020) [28] proposed the square wave (SW) approach, where values close to the true values are published with high probability while values far from the true values are published with low probability, thereby estimating the distribution of one-dimensional numerical attributes. Duchi et al. [6, 7] also based on random response mechanism, proposed the MeanEst method for mean value estimation, with high data usability but not applicable to high dimension dataset. Subsequently, Nguyên et al. [8] optimized the scheme of Duchi et al. and proposed the Harmony algorithm for collecting and analyzing data from smart devices, which can support both frequency and mean value estimation as well as machine learning tasks with high data usability. Wang et al. (2019) [29] further proposed the Piecewise Mechanism (PM) for handling frequency and mean value estimation with a high usability, but computationally complex and difficult to encode. All the above methods are studies on non-streaming data, i.e., one-dimensional or multi-dimensional attribute individual data for statistics, and do not involve the study of one-dimensional or multi-dimensional attribute stream data.

The above privacy protection techniques for wearable device data collection are summarized in Table 1.

**Table 1:** Classification of privacy protection technology for wearable device data.

Categories	Characteristics	Technologies	Representative Papers
Cryptography-based schemes	High security, but low efficiency	Homomorphic encryption	Wei et al. (2021) [20]
		Secure multi-party computation	Hua et al. (2018) [22]
Perturbation-based schemes	High calculation efficiency, but data accuracy can be biased	Anonymization	Liu et al. (2018) [24]
		Centered differential privacy	Yang et al. (2020) [27]
		Local differential Privacy	Li et al. (2020) [28]
			Wang et al. (2019) [29]

Stream data collection for local differential privacy, on the one hand, has been studied in terms of frequency estimation. Ning Guo (2019) [30] collected data from users’ browsing on the scenario of web page click ranking with a mixture of centered differential privacy and local differential privacy. Afrose et al. (2021) [31] proposed local differential privacy for stream data using RAPPOR technique to obtain frequency estimation. Arcolezi et al. (2021) [32] conducted a study on temporal data of multidimensional attribute using three state-of-the-art protocols. Wang et al. (2021) [33] proposed a method to determine the optimal threshold with exponential mechanism to satisfy the data stream publishing problems. A hierarchical approach was used to divide the data streams and add proportional noise to the threshold of each layer, which can satisfy the range query but cannot be refined to an estimate at a certain moment. The above are all frequency estimation or range queries for stream data, and the mean value estimation for stream data are not studied.

On the other hand, some scholars have studied it in the aspect of mean statistics. Kellaris et al. [9] proposed w-event privacy mechanism based on centered differential privacy, which gives a solution to the issue of allocating the privacy budget of stream data. It is divided into budget absorption (BA) and budget distribution (BD) schemes, where the former reuses the previously unallocated privacy budget and the latter allocates the privacy budget in an exponentially decreasing manner, but for each sliding window, half of the privacy budget is fixedly consumed before further allocation of the remaining budget, which leads to the waste of the privacy budget and makes the data usability decrease and affects the overall accuracy. Errounda et al. [10] applied w-event privacy mechanism for location statistics based on local differential privacy and used an approximation strategy to estimate the unperturbed locations, but similarly may incur unnecessary utility loss. Fang et al. [11] proposed local differential Private Streaming Protocol (LDPS), based on local differential privacy with w-event privacy mechanism. It can adaptively invoke methods to handle categorical and numerical data, but the resource consumption is too large to be applicable to wearable devices whose resources are limited.

For stream data generated by wearable devices, Kim et al. [13] proposed a new idea to remove the adjacent data with the same value, or with the same trend, get the salient points and then use the Laplace mechanism to add noise. Finally, the stream data graph is reconstructed according to a straight line or curve, which reduces the unnecessary consumption of privacy budget and increases the usability of the data. Kim et al. (2019) [34] continued their study in this area by applying the previous method to smart watches and collected data in the form of histograms and streaming data. Kim et al. (2020) [35] continually improved the optimization of the method for identifying salient points, allowing it to be applied to more possible stream data scenarios. However, the issue of data usability aspect remains problematic.

In short, the current study on local differential privacy for stream data is not comprehensive and mainly focuses on frequency estimation. For mean value estimation, the existing solutions still suffer from excessive resource consumption or poor data usability.

## Wearable device data protection based on local differential privacy

### Theoretical basis

#### Local differential privacy

Local differential privacy [36] is an extension of centered differential privacy, in which centered differential privacy aggregates users’ data to the service provider (i.e., a third party) and then allows the service provider to perturb the data before analyzing and using it; local differential privacy, in contrast, puts the work of perturbing data at users’ devices, where the users send the perturbed data to the service provider for analysis and use. A formal definition of local differential privacy [3] is as follows:

Given *n* users, each user corresponds to one record, given a privacy algorithm *M* and its definition domain *Dom*(*M*) and value domain *Ran*(*M*), if the algorithm *M* inputs any two records *t* and *t*′(*t*, *t*′ ∈ *Dom*(*M*)) to obtain the same output result t*(t* ∈ *Ran*(*M*)), satisfying the following inequality, then *M* satisfies *ϵ*-local differential privacy.

$$\begin{array}{c} Pr\left[M\left(t\right)=t^{*}\right]\leq e^{\epsilon }\cdot Pr\left[M\left(t^{\prime }\right)=t^{*}\right] \end{array}$$ (1)

Where *Pr*[⋅] denotes the probability of privacy being disclosed; *ϵ* is the privacy budget, which is used to control the ratio of the output probability of algorithm *M* on two records and reflects the level of privacy protection that algorithm *M* can provide. A larger privacy budget means higher data usability and lower security. Conversely, a smaller privacy budget means lower data usability and higher security.

#### Laplace mechanism

Laplace mechanism [37] is a very classical noise addition method in centered differential privacy, which can also be applied to local differential privacy. It is described as follows:

Assuming that each user *u*_*i*_ records a numerical attribute value *t*_*i*_, and there are *n* users in total. Defining the following random function to add noise to *t*_*i*_.

$$\begin{array}{c} t_{i}^{*}=t_{i}+Lap\left(\frac{\Delta s}{\epsilon }\right) \end{array}$$ (2)

Where $t_{i}^{*}$ is the value with the noise addition and *Lap*(λ) represents the random variable that follows the Laplace distribution of scale $\lambda (\lambda =\frac{\Delta s}{\epsilon })$, i.e., noise. Δ*s* is the difference between the maximum value and the minimum value in attribute *t*_*i*_. The Laplace mechanism has the following probability density function: $pdf\left(x\right)=\frac{1}{2\lambda }exp\left(-\frac{\left|x\right|}{\lambda }\right)$ (the probability density function of the Laplace distribution with expectation of 0 and variance of 2λ^2^). Clearly, the estimate $t_{i}^{*}$ is unbiased, since the injected Laplacian noise $Lap(\frac{\Delta s}{\epsilon })$ in each $t_{i}^{*}$ has zero mean (i.e., expectation of 0). Unbiasedness means that the mathematical expectation of the estimator is equal to the true value of the parameter being estimated, and it is a criterion used to evaluate the goodness of the estimator.

Each user adds a $Lap(\frac{\Delta s}{\epsilon })$ of Laplace noise to the data and sends it to the data collector, who averages the resulting tuple of data $\left(\frac{1}{n}\sum_{i=1}^{n}t_{i}^{*}\right)$ to obtain the statistical value of the attribute with a probability error of $O(\frac{1}{\epsilon \sqrt{n}})$.

#### MeanEst method

The idea of the MeanEst method [6] is to perturb a given data value to one of two fixed values by random response. Assuming a given tuple *y*_*i*_, *y*_*i*_ ∈ [−1, 1], which represents the value of an attribute of *i* users, the value of user one is *y*_1_, the value of user two is *y*_2_, and the value of user *i* is *y*_*i*_.

$$\begin{array}{c} Pe\left[y_{i}^{*}=t|y_{i}\right]=\{\begin{array}{c} \frac{e^{\epsilon }-1}{2e^{\epsilon }+2}\cdot y_{i}+\frac{1}{2},\;t=\frac{e^{\epsilon }+1}{e^{\epsilon }-1}\\ -\frac{e^{\epsilon }-1}{2e^{\epsilon }+2}\cdot y_{i}+\frac{1}{2},\;t=-\frac{e^{\epsilon }+1}{e^{\epsilon }-1} \end{array} \end{array}$$ (3)

With probability according to Eq (3), they are perturbed to $y_{i}^{*}$, i.e., perturbed to $\frac{e^{\epsilon }+1}{e^{\epsilon }-1}$ or $-\frac{e^{\epsilon }+1}{e^{\epsilon }-1}$, where *Pe*[⋅] is the perturbation probability and *t* is the value after perturbation.

### The approach of data collection and protection based on local differential privacy

#### Overall architecture for wearable device data collection

**Fig 1:**
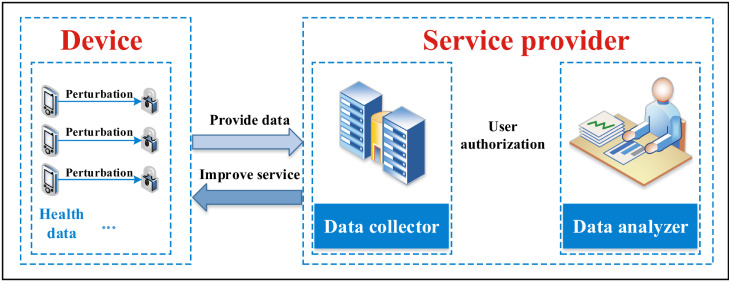
Overall architecture of data collection for wearable devices.

The overall architecture of data collection for wearable devices is shown in Fig 1, which is divided into two entities: Device and Service provider. The device collects data and perturbs it locally, and then sends the perturbed data to the service provider. The service provider is divided into a data collector and a data analyzer. The data collector reconstructs the perturbed data and holds it for later use. After all data are processed, the data analyzer is authorized by the user to conduct statistical analysis. The service provider performs product optimization and improves the service to the user based on the collected information.

**Fig 2:**
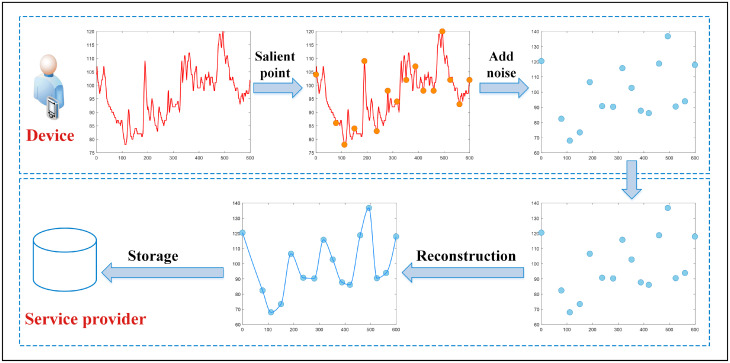
Data collection privacy protection process based on local differential privacy.

The specific process of local differential privacy protection for the stream data generated by each wearable device is shown in Fig 2. Following the idea provided by Kim et al. [13], it is also divided into device and service provider.

- Device: The wearable device collects data at a fixed time interval, and collects the data after a period of time so as to form a curve graph. Before adding noise, the data needs to be processed and only part of data is added noise instead of all data for reducing the consumption of privacy budget. For that purpose, it is necessary to delete the redundant data and identify which are the salient points, i.e., the points that represent the graph best. After the salient points are identified, noise is added to the salient points by adopting an adaptive Laplace mechanism and finally the noised data is sent to the service provider.
- Service provider: The service provider receives the noise-added data, reconstructs them in different ways to reconnect the discrete points, calculates the missing data values, and stores them for backup after reconstructing. When the data of all devices are processed, the statistical results are generated.

#### Privacy protection for wearable device data collection

1 Data acquisition.

The wearable device collects data at fixed intervals, and records the time stamp *t*_*i*_ and the corresponding data *x*_*i*_ each time, 0 < *i* ≤ *n*, *n* is the length of the time stream, which eventually forms the data stream of the device. Assuming that the device is *s*_1_, the data it collects is *s*_1_ = (*t*_1_, *x*_1_), (*t*_2_, *x*_2_), …, (*t*_*n*_, *x*_*n*_). Wearable devices can customize the sampling interval according to the attribute characteristics because of the different attributes of the data they collect. For example, heart rate data is collected every minute and blood oxygen data is collected every hour.

Simply connecting the collected time series data can form a graph of the collected health data.

2 Identification of salient points.

The salient points are the one that represent the curve trend best, so it is necessary to use the nature of the derivative, i.e., a positive (negative) derivative means a straight line up (down) and a derivative of 0 stands for a straight line level, so as to achieve the purpose of identifying the salient points. The equation for calculating the derivative of each data point is as follows:

$$\begin{array}{c} d_{i}=\frac{x_{i}-x_{i-1}}{t_{i}-t_{i-1}} \end{array}$$ (4)

Where *d*_*i*_ denotes the derivative of point *i*, points (*t*_*i*−1_, *x*_*i*−1_), (*t*_*i*_, *x*_*i*_) are two adjacent points, *t*_*i*_ denotes the moment *i*, *x*_*i*_ denotes the data value collected at moment *i*, and *t*_*i*−1_ and *x*_*i*−1_ likewise.

Identifying salient points is a process of removing redundancy from existing data points. It will represent the entire curve with a smaller number of points so as to reduce the consumption of privacy budget. In short, there are three types of redundant data points, the first is the points with equal data within consecutive timestamps, the second is the points with a consistent trend within a period of time, and the third is the points with frequent fluctuations in certain time periods. The first two types are discriminated by using derivatives of zero and derivatives of the same sign, respectively, while the last type specifies the maximum interval time as a threshold for judgment.

First, after obtaining the derivatives of all data points, we find and remove the data points with zero derivatives (indicating that the data values of the two points are equal). Second, on this basis, we traverse the remaining data points from the beginning, keeping the first point and regarding this point as the starting point, where the derivatives of adjacent data points with the same sign indicate the same trend between them, then we continue to traverse backward until we encounter the turning point (the first point when the sign of the derivatives of adjacent data points is opposite, and the time interval from the starting point is greater than threshold *α*). Third, we keep the turning point and delete the intermediate data points from the starting point to this point, and repeat the above steps with the turning point as the new starting point. Until we traverse to the last point, which is taken as the last turning point, repeating the previous steps. Finally, the remaining points are the salient points.

Algorithm 1 gives the pseudo-code for identifying salient points. Given the stream data, first, we calculate the derivatives of the two adjacent points, remove all the points with 0 derivatives, and store the points that are not deleted in *list*1 (lines 1–9). Second, we process the remaining points, traverse *list*1 from front to back. If the derivatives of the adjacent data points have the same sign (i.e., same positive or negative), we delete the former and continue to compare the latter with the next data point. If the derivatives of adjacent data points are opposite, and the time interval (*t*_*int*_) between the former point and the starting point (i.e., *t*_*cur*_ and *t*_*sta*_ respectively) is greater than *α*, the former is kept and the latter is compared with the next data point. Repeat the previous steps until the *list*1 is traversed, and the retained points together with the starting and ending points are the salient points, which are stored in *list*2 (lines 10–22).

**Algorithm 1** Identification of salient points

**Input**: (*t*_1_, *x*_1_), (*t*_2_, *x*_2_), …, (*t*_*n*_, *x*_*n*_), *α*

**Output**: (*t*_1_, *x*_1_), (*t*_*a*_, *x*_*a*_), …, (*t*_*n*_, *x*_*n*_)

1: *i* = 1, (*t*_0_, *x*_0_) = (0, 0);

2: **for** 1: *n* **do** // Traversing stream data

3:  $d_{i}=\frac{x_{i}-x_{i-1}}{t_{i}-t_{i-1}}$;

4:  **if** *d*_*i*_ = 0 **then**

5:   Delete (*t*_*i*_, *x*_*i*_);

6:  **else**

7:   (*t*_*i*_, *x*_*i*_) ∈ *list*1

8:  **enf if**

9: **end for**

10: (*t*_0_, *x*_0_) ∈ *list*2, *j* = 2, *p* = *count*(*list*1), *t*_*sta*_ = 0;

11: **for** *j*: *p* **do** // Traversing *list*1

12:  *t*_*int*_ = *t*_*cur*_ − *t*_*sta*_;

13:  **if** (*d*_*j*_ > 0&&*d*_*j*+1_ > 0) ∥ (*d*_*j*_ < 0&&*d*_*j*+1_ < 0) **then**

14:   Delete (*t*_*j*_, *x*_*j*_);

15:  **else if** *t*_*int*_ ≤ *α* **then**

16:   Delete (*t*_*j*_, *x*_*j*_);

17:  **else**

18:   (*t*_*j*_, *x*_*j*_) ∈ *list*2;

19:   *t*_*sta*_ = *t*_*cur*_;

20:  **end if**

21:  (*t*_*n*_, *x*_*n*_) ∈ *list*2

22: **end for**

3 Adding noise to data.

In this study, we multiply the original Laplacian mechanism of Dwork et al. [37] by an adaptive random value *r*_*i*_, which is called the adaptive Laplacian mechanism, and we use it to add noise to the salient points, shown in Eqs (5), (6) & (7).

First, given the tuple *x*_*i*_, we normalize it to the tuple *y*_*i*_, *y*_*i*_ ∈ [−1, 1], according to Eq (5).

$$\begin{array}{c} y_{i}=\frac{x_{i}-x_{mean}}{x_{\text{max}}-x_{\text{min}}} \end{array}$$ (5)

Where *x*_*i*_ is the value corresponding to moment *i*, *x*_*min*_, *x*_*max*_, *x*_*mean*_ are the minimum, maximum and mean values of the data set respectively, and *y*_*i*_ is the value corresponding to *x*_*i*_ after normalizing to [−1, 1].

Then, the random value *r*_*i*_ corresponding to each moment is calculated according to Eq (6). The random value refers to the idea of random response in MeanEst algorithm of Duchi et al [6], and the normalized value *y*_*i*_ at moment *i* is transformed into the corresponding value *r*_*i*_ according to the perturbation probability. Since the numerical size of the random value *r*_*i*_ depends on the size of *x*_*i*_ at this moment, we call it an adaptive random value.

Finally, according to Eq (7), the *x*_*i*_ at each moment is noise-added.

$$\begin{array}{c} r_{i}=\frac{e^{\epsilon }-1}{2e^{\epsilon }+2}\cdot y_{i}+\frac{1}{2} \end{array}$$ (6)

$$\begin{array}{c} x_{i}^{*}=x_{i}+r_{i}\cdot Lap\left(\frac{\Delta s}{\epsilon_{i}}\right) \end{array}$$ (7)

Where *r*_*i*_ is the random value, *ϵ* is the privacy budget, *y*_*i*_ is the value corresponding to *x*_*i*_ normalized to [−1, 1], $x_{i}^{*}$ is the value after noise addition, *x*_*i*_ is the value recorded at this moment, Δ*s* is the difference between the maximum and minimum values in the tuple *x*_*i*_, and *ϵ*_*i*_ is the privacy budget assigned at this moment.

Since the random value *r*_*i*_ (0 ≤ *r*_*i*_ ≤ 1) is calculated based on the normalized data *x*_*i*_, it is perturbation adaptive for the given data. The multiplication by the Laplace function adaptively reduces the size of the added perturbation noise value and increases the usability of the data.

Proof: The random value 0 ≤ *r*_*i*_ ≤ 1.

Let $f\left(\epsilon \right)=\frac{e^{\epsilon }-1}{2e^{\epsilon }+2}$, $r_{i}=f\left(\epsilon \right)\cdot y_{i}+\frac{1}{2}$, *ϵ* ≥ 0. Since $f^{{}^{\prime }}\left(\epsilon \right)=\frac{4e^{\epsilon }}{{\left(2e^{\epsilon }+2\right)}^{2}}>0$ and *f*(0) = 0, so *f*(*ϵ*) ≥ 0 and monotonically increasing. Since ${\text{lim}}_{\epsilon \to +\infty }f\left(\epsilon \right)={\text{lim}}_{\epsilon \to +\infty }\frac{e^{\epsilon }-1}{2e^{\epsilon }+2}=\frac{1}{2}$, so $0\leq f\left(\epsilon \right)\leq \frac{1}{2}$. And since *y*_*i*_ ∈ [−1, 1], *f*(*ϵ*) takes the maximum value $\frac{1}{2}$, it is known that the range of *r*_*i*_ is [0, 1], i.e., 0 ≤ *r*_*i*_ ≤ 1.

According to the Laplace mechanism, the data after noise addition by using the Laplace function is unbiased, and the values after adding noise to the data by Eq (7) above are also unbiased (i.e., each $x_{i}^{*}$ is unbiased).

Proof: Since the mathematical expectation of the inserted noise $Lap(\frac{\Delta s}{\epsilon_{i}})$ is zero and *r*_*i*_ is a constant, $E\left(x_{i}^{*}\right)=E\left(x_{i}+r_{i}\cdot Lap\left(\frac{\Delta s}{\epsilon_{i}}\right)\right)=x_{i}+r_{i}\cdot 0=x_{i}$, where the mathematical expectation of the estimator is equal to the true value of the estimated parameter, so it is unbiased.

Algorithm 2 gives the pseudo-code to compute the adaptive random values, taking the previously determined salient points, i.e., *list*2, as data input. This set of data is normalized to [−1, 1], and then the random values are calculated sequentially and stored in *list*3.

**Algorithm 2** Adaptive random values

**Input**: *x*_1_, *x*_2_, …, *x*_*p*_ *ϵ x*_*i*_ ∈ [*x*_*min*_, *x*_*max*_]

**Output**: *r*_1_, *r*_2_, …, *r*_*p*_ *r*_*i*_ ∈ [0, 1]

1: Find the minimum, maximum and mean values from *list*2;

2: **for** *i*: *p* **do**

3:  $y_{i}=\frac{x_{i}-x_{mean}}{x_{max}-x_{min}}$;

4:  $r_{i}=\frac{e^{\epsilon }-1}{2e^{\epsilon }+2}\cdot y_{i}+\frac{1}{2}$;

5:  *r*_*i*_ ∈ *list*3;

6: **end for**

Algorithm 3 gives the pseudo-code for the noise addition with the adaptive Laplacian mechanism. The total privacy budget is *ϵ*, and the privacy budget is equally divided for the remaining *p* salient points, i.e., the privacy budget for each data is $\epsilon_{i}=\frac{\epsilon }{p}$. Input the processed data, *list*2 with *list*3 and the privacy budget *ϵ*_*i*_, and apply the adaptive Laplace algorithm to each original data in turn to add noise and store it in *list*4.

**Algorithm 3** Adaptive Laplacian mechanism

**Input**: (*t*_1_, *x*_1_), (*t*_*a*_, *x*_*a*_), …, (*t*_*n*_, *x*_*n*_)*r*_1_, *r*_*a*_, …, *r*_*n*_ *ϵ*_1_, *ϵ*_*a*_, …, *ϵ*_*n*_

**Output**: $\left(t_{1},x_{1}^{*}\right),\left(t_{a},x_{a}^{*}\right),\ldots ,\left(t_{n},x_{n}^{*}\right)$

1: **for** *i*: *p* **do**

2:  $x_{i}^{*}=x_{i}+r_{i}\cdot Lap\left(\frac{\Delta s}{\epsilon_{i}}\right)$;

3:  $x_{i}^{*}\in list4$;

4: **end for**

4 Data reconstruction.

The data collector receives noisy data from each user and reconstructs the data for each of these users separately to calculate the mean value at each moment. The essence of reconstruction is to concatenate discrete data points to calculate the value of missing data points. Different connection methods have different data errors, so this study uses three different reconstruction methods to determine which method has the lowest error and improves data usability. These three approaches are linear, pchip and spline [38].

Linear is the simplest way to connect the discrete points by straight lines directly in sequence, and the missing data are found on the function of the corresponding straight line. The reconstructed curve looks very unsmooth in general. The expression of the function for each subinterval is as follows:

$$\begin{array}{c} \begin{array}{c} L\left(x\right)=y_{k}+\left(x-x_{k}\right)\frac{y_{k+1}-y_{k}}{x_{k+1}-x_{k}}\\ x\in [x_{k},x_{k+1}],k=1,2,\ldots ,n-1 \end{array} \end{array}$$ (8)

Where (*x*_*k*_, *y*_*k*_), (*x*_*k*+1_, *y*_*k*+1_) are adjacent discrete points, which are known points, and the missing *y* value data between the two points are completed according to the function of each subinterval.

Pchip is called piecewise cubic Hermite interpolating polynomial, which needs to satisfy the first-order derivative continuous and monotonically varying in each interval, i.e., the first-order derivative value of each point is set as the weighted harmonic mean of the slopes of the left and right sides of the cut line. This makes the curve smooth and conformal at the same time. Suppose there are two points (*x*_0_, *y*_0_), (*x*_1_, *y*_1_), *x*_0_ < *x*_1_ and assume that the first order derivatives exist and are *d*_0_ and *d*_1_ respectively. let $h=x_{1}-x_{0},s=x-x_{0},\delta =\frac{y_{1}-y_{0}}{x_{1}-x_{0}}$. The function of pchip and first order derivatives are shown below.

$$\begin{array}{c} \begin{array}{cc} P(x) & =\frac{h^{3}-3hs^{2}+2s^{3}}{h^{3}}y_{0}+\frac{3hs^{2}-2s^{3}}{h^{3}}y_{1}+\frac{s{\left(s-h\right)}^{2}}{h^{2}}d_{0}+\frac{s^{2}\left(s-h\right)}{h^{2}}d_{1}\\ & =y_{0}+sd_{0}+\frac{s^{2}\left(3\delta -2d_{0}-d_{1}\right)}{h}+\frac{s^{3}\left(d_{0}-2\delta +d_{1}\right)}{h^{2}} \end{array} \end{array}$$ (9)

$$\begin{array}{c} P^{{}^{\prime }}\left(x\right)=d_{0}+\frac{2s(3\delta -2d_{0}-d_{1})}{h}+\frac{3s^{2}\left(d_{0}-2\delta +d_{1}\right)}{h^{2}} \end{array}$$ (10)

Spline is called cubic spline interpolation, which has the same function with pchip, i.e., Eq (9), but it is more strict and requires the second order derivative to be continuous, where the second order derivative is shown in Eq (11). Therefore, spline is smoother than pchip, but not necessarily shape preserving.

$$\begin{array}{c} P^{{}^{\prime \prime }}\left(x\right)=\frac{\left(6h-12s\right)\delta +\left(6s-2h\right)d_{1}+\left(6s-4h\right)d_{0}}{h^{2}} \end{array}$$ (11)

5 Data collection and analysis.

The data collector completes the data collection by forming a data set based on the collection cycle for reconstructing the curve. The data analyst analyzes the resulting data set, calculating the mean value of all devices at each moment, and generating statistics that are aggregated and analyzed to improve the product and optimize the user experience.

The related information is shown in Table 2. The original dataset of all devices is *D*, the dataset of one device is *d*_*i*_, the dataset of all devices after adding noise is D*, and the reconstructed dataset of one device is denoted by $d_{i}^{*}$.

**Table 2:** Related information summary.

Descriptions	Notations
Raw dataset for all devices	*D* = {*d*_1_, *d*_2_, …, *d*_*w*_}
Single device datasets	*d*_*i*_ = ((*t*_1_, *x*_1_), (*t*_2_, *x*_2_), …, (*t*_*n*_, *x*_*n*_)) *i* ∈ [1, *w*]
Noise added dataset for all devices	$D^{*}=\left\{d_{1}^{*},d_{2}^{*},\ldots ,d_{w}^{*}\right\}$
Single device reconstructed datasets	$d_{i}^{*}=\left(\left(t_{1},x_{1}^{*}\right),\left(t_{2},x_{2}^{*}\right),\ldots ,\left(t_{n},x_{n}^{*}\right)\right)$ *i* ∈ [1, *w*]

We aggregate the data of all devices ($d_{1}^{*}∼d_{w}^{*}$) at each moment (*t*_1_ ∼ *t*_*n*_) and calculate the mean value corresponding to moment *t*_*i*_ as the estimated value of this moment using Eq (12), i.e., *AVG*_*est*_(*x*_*i*_). According to this method, the estimated values corresponding to all moments are calculated.

$$\begin{array}{c} AVG_{est}\left(x_{i}\right)=\frac{1}{w}\times \sum_{d_{1}^{*}}^{d_{w}^{*}}x_{i}^{*} \end{array}$$ (12)

Where *w* denotes that there are *w* devices collecting data, and $x_{i}^{*}$ denotes the value after adding noise at moment *t*_*i*_. The expected error of the mean is $O(\frac{n}{\epsilon \sqrt{w}})$, where *n* denotes the number of attributes that need to be noised (corresponding to the number of salient points in this study), *ϵ* denotes the privacy budget, *w* denotes the number of users (corresponding to the number of devices in this study). The error is proportional to the size of *n*, so the larger *n* is, the higher error will be.

### Evaluation indicators for the privacy protection of wearable device data

In order to determine the most suitable reconstruction method and to evaluate the experimental results of our scheme and the reference scheme, Mean Relative Error (MRE) and Root Mean Square Error (RMSE) are used as evaluation indicators in this study.

$$\begin{array}{c} MRE=\frac{1}{n}\times \sum_{i=1}^{n}\frac{\left|AVG_{actual}\left(x_{i}\right)-AVG_{est}\left(x_{i}\right)\right|}{AVG_{actual}\left(x_{i}\right)} \end{array}$$ (13)

$$\begin{array}{c} RMSE=\sqrt{\frac{1}{n}\times \sum_{i=1}^{n}{\left(AVG_{actual}\left(x_{i}\right)-AVG_{est}\left(x_{i}\right)\right)}^{2}} \end{array}$$ (14)

Where *n* denotes the length of the stream data, *i* denotes the i-th moment, *x*_*i*_ denotes the value collected at the i-th moment, *AVG*_*actual*_(*x*_*i*_) is the mean value of the corresponding actual data at *t*_*i*_, and *AVG*_*est*_(*x*_*i*_) is the mean value of the corresponding estimated data at *t*_*i*_.

These two indicators are the standard to measure the change range of a variable, for example, in the same amount of data, as the privacy budget keeps increasing and the error varies with it. Among them, RMSE is more sensitive to higher error values because each error is calculated as a square.

We use the MRE to measure the usability of the data, where a smaller value indicates a smaller error and higher usability. RMSE is used to measure thebias of data, where a smaller value indicates less bias in the data.

## Experiment and analysis

### Experimental dataset

In this study, experiments are conducted under the real dataset, the PAMAP2 dataset [13, 39]. The dataset contains 9 testers’ tested data under 18 activity items. We select 8 testers’ heart rate data as the data for this experiment (the other one has incomplete data), while the testers are numbered from 101 to 108. Every tester’s data of heart rate is collected once per minute, and 3000 heart rate data are collected in total for each tester. So the total data is 24K (8*3000). Considering the power of the wearable device and the user’s personal reasons, the user usually not use the wearable device for 50 hours (i.e., 50 hours to collect 3000 records). Meanwhile, in order to maintain the overall characteristics of the dataset, we condense the data of each tester to the amount of 10 hours of collection in a day, i.e., we take the first record of every five of 3000 records which will be condensed to 600 records. The heart rate data of the testers are shown in Table 3.

**Table 3:** Dataset summary.

Dataset number	101	102	103	104	105	106	107	108
Number of instances	600	600	600	600	600	600	600	600
Value range	78∼120	74∼107	68∼94	57∼121	70∼101	60∼104	60∼99	66∼104

We replicate each tester’s data to form total datasets of 120K, 240K, 360K, 480K and 600K so as to explore the impact of data sizes on data usability.

### Experimental methods

We adopt the controlled variable method in this experiment, and we get the experimental data from different reconstruction methods (linear, pchip, spline), different privacy budgets *ϵ* (0.5, 1, 2) and different data sizes (120K, 240K, 360K, 480K, 600K). Firstly, we compare the estimated values with the actual values according to different reconstruction methods. Secondly, the two indicators of MRE and RMSE are calculated (Eqs 13 & 14) to determine the reconstruction method with the lowest error. Finally, we use the lowest error reconstruction method for experiments and compare it with the reference schemes. Then we calculate the two indicators of MRE and RMSE so as to judge the performance of our scheme.

Our scheme first identifies the salient points (parameter *α* is set to 30), adds noise, and reconstructs the data for each user respectively. Then, we aggregate the data of all users according to the reconstruction curve and calculate the mean value at each moment. Finally, we compare it with the mean value of each moment before noise addition for all users and calculate the two indicators of MRE and RMSE. To reduce occasional errors, we repeat the experiment several times to take the average value as the experimental results.

Our experiments are run on a Windows 10 machine using Python 3.9 and MATLAB 2020b with 16G RAM and AMD Ryzen 5 5600H CPU.

### Experimental results and discussion

#### Impact of privacy budgets and data sizes between actual and estimated values

**Fig 3:**
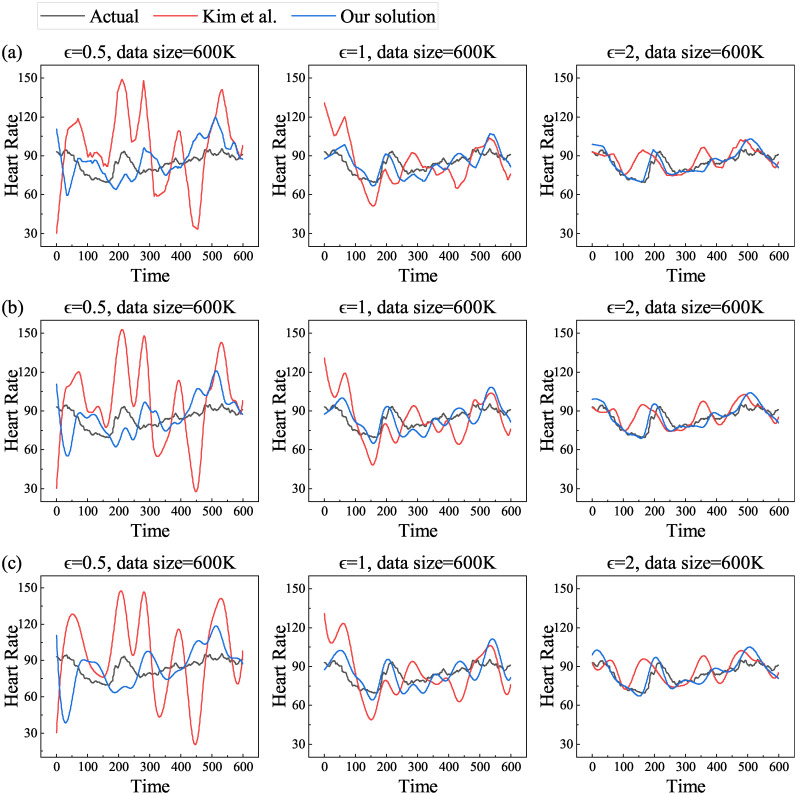
Actual and estimated values of mean heart rate with increasing privacy budget for different reconstruction methods. (a) linear, (b) pchip and (c) spline.

In order to study the relationship between privacy budget sizes and actual and estimated values, we select a dataset with data size of 600K, privacy budget *ϵ* is taken as 0.5, 1, and 2, respectively, and the linear, pchip, and spline methods are in turn taken for reconstructing the curve. We compare the scheme of Kim et al. [13] (the same below) and calculate the mean value for each moment. The results are shown in Fig 3.

From the above figure, we can see that the estimated value matches the actual value more and more as the privacy budget *ϵ* keeps increasing, regardless of the noise addition method as well as the reconstruction method. This is because the size of the privacy budget is inversely related to the size of the noise. The smaller privacy budget is, the higher degree of privacy protection will be, and therefore the noise added will be larger, which is reflected from the graph that the estimated value is more deviated from the actual value. Conversely, the larger privacy budget is, the lower degree of privacy protection will be, and therefore the noise added will be smaller, which means the estimated value is closer to the actual value. From the three different reconstruction methods, the data graphs reconstructed by linear method are not smooth, while pchip and spline methods are obviously smooth, as well as the gap between the actual and estimated values varies.

**Fig 4:**
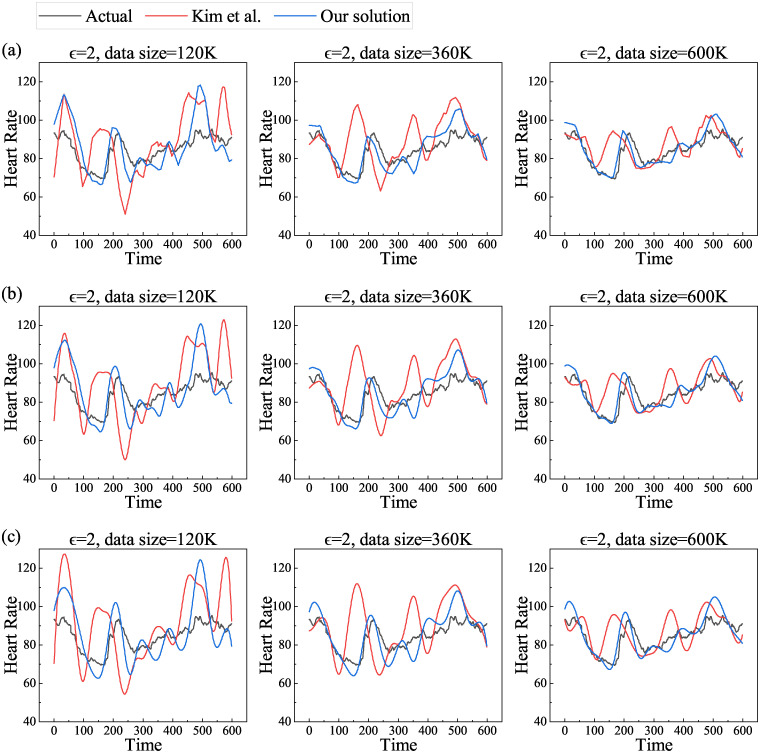
Actual and estimated values of mean heart rate with increasing data size for different reconstruction methods. (a) linear, (b) pchip and (c) spline.

In order to study the impact of data sizes on actual and estimated values, we first unify the privacy budget to 2, and the datasets are taken to be 120K, 360K, and 600K respectively. Then we choose linear, pchip and spline methods in turn to reconstruct the curve and calculate the mean value at each moment. The results are shown in Fig 4.

From the Fig 4, we can see that the estimated value matches the actual value with increasing amount of data, regardless of the noise-addition scheme and the reconstruction method. This is because the expected error of both Kim et al.’s scheme and our scheme is $O(\frac{n}{\epsilon \sqrt{w}})$(*n* denotes the number of attributes to be noise-added, in this study denotes the number of salient points, *ϵ* denotes the privacy budget, and w denotes the number of users), which decreases as the number of users increases, i.e., the estimated value is closer to the actual value as the amount of data increasing. Similarly, the data characteristics under these three reconstructions are the same as the experiments under different privacy budgets above.

#### Impact of different reconstruction methods on MRE and RMSE

**Fig 5:**
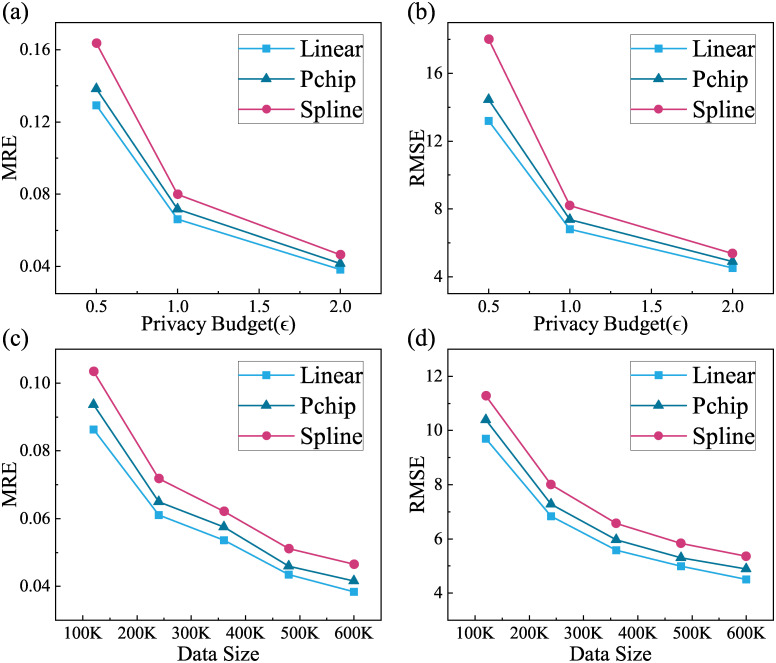
Errors under different reconstruction methods. (a) MRE under different privacy budgets, (b) RMSE under different privacy budgets, (c) MRE under different data sizes and (d) RMSE under different data sizes.

For the purpose of studying the impact of different reconstruction methods on MRE and RMSE, we need to conduct experiments on privacy budget and data size separately, i.e., under the same dataset (600K) or uniform privacy budget (*ϵ* = 2), so as to find out which method has the smallest error under different reconstruction methods. This experiment uses our scheme for the noise addition, and the experimental results are shown in Fig 5.

As we can see from Fig 5, the MRE and RMSE of the reconstruction with the linear and pchip methods are both lower than that of spline method, and the reconstruction error with the spline method is slightly larger (reconstruction curves are shown in the third column of Figs 3 or 4). Among them, the difference between the indicator values of linear and pchip is very small, and the specific data are shown in Tables 4 and 5.

**Table 4:** Errors under different privacy budgets.

	MRE	MRE	MRE	RMSE	RMSE	RMSE
Privacy budget	0.5	1	2	0.5	1	2
Linear	**0.1292**	**0.0662**	**0.0383**	**13.2004**	**6.7997**	**4.5037**
Pchip	0.1383	0.0717	0.0416	14.4537	7.3646	4.8847
Spline	0.1635	0.0799	0.0466	18.0156	8.1994	5.3600

**Table 5:** Errors under different data sizes.

	MRE	MRE	MRE	RMSE	RMSE	RMSE
Data Size	120K	360K	600K	120K	360K	600K
Linear	**0.0863**	**0.0537**	**0.0383**	**9.6957**	**5.5881**	**4.5037**
Pchip	0.0936	0.0575	0.0416	10.3896	5.9640	4.8847
Spline	0.1033	0.0621	0.0466	11.2769	6.5701	5.3600

In both calculations of the indicators, the linear method is slightly better than the pchip one. The reason is that noise added data deviates from the original value and the reconstruction curve with the spline method is more smoothly. It will deviate from the actual value a bit further compared with that of linear method, so its error is larger than the other two. The reconstruction with pchip method is partial to linear method, so from the results, the errors are similar to the linear method.

Therefore, the reconstruction with the linear method is the one with the least error in this study.

#### Impact of privacy budgets on MRE and RMSE

**Fig 6:**
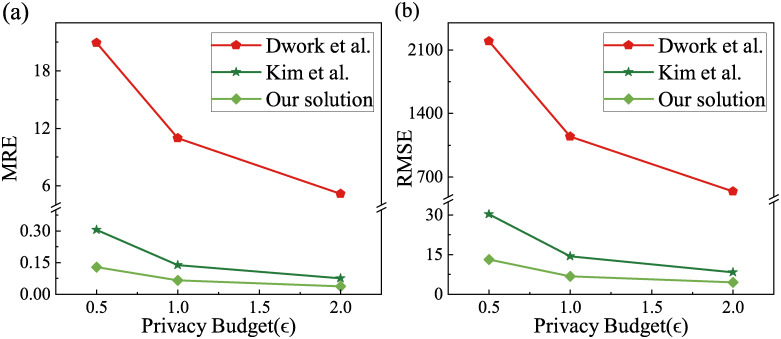
Errors under different privacy budgets. (a) MRE of three algorithms and (b) RMSE of three algorithms.

We select a dataset with a data size of 600K and privacy budgets *ϵ* of 0.5, 1, 2 for the experiments and calculate the MRE and RMSE in order to study the impact of privacy budgets on the two evaluation indicators. The reconstruction uses the linear method and compares with the schemes of Dwork et al. [37] and Kim et al. [13] (the same below). The results are shown in Fig 6.

In Fig 6, we can see that, for the results of the two indicators calculation, the error of Dwork et al.’s scheme is much larger than Kim et al.’s and our scheme. Moreover, the error of our scheme is lower than Kim et al.’s scheme in both indicators, which means that the data usability and data bias of our scheme are better than those of comparison schemes. As the privacy budget increases, both MRE and RMSE of three solutions become smaller and smaller. The reason is that the privacy budget essentially represents the degree of protection of user privacy, which has been explained in the previous section on “the relationship between privacy budget sizes and actual and estimated values” and will not be repeated here. Table 6 shows the specific data of the above MRE under different privacy budgets (Fig 6(a)), where the decline rate is the reduction of MRE for our scheme compared to Kim et al.’s scheme (the same below).

**Table 6:** MRE under different privacy budgets.

Privacy budget	Dwork et al.	Kim et al.	Our solution	Decline rate
0.5	20.9119	0.3060	0.1292	57.78%
1	10.9796	0.1391	0.0662	52.41%
2	5.1945	0.0759	0.0383	49.54%

It can be seen that our scheme reduces the MRE by 57.78% compared to Kim et al.’s scheme at a privacy budget of 0.5, i.e., a higher privacy protection strength, suggesting that the usability of our data is better than the former one by over a half.

So far, we can conclude from these two indicators that the scheme of our work is better than Kim et al.’s scheme under different privacy budgets.

#### Impact of data sizes on MRE and RMSE

**Fig 7:**
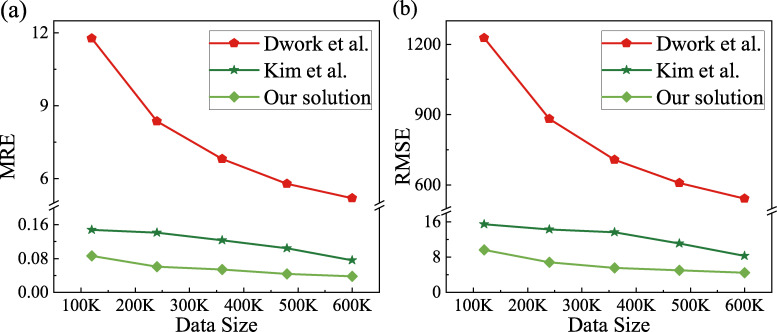
Errors under different data sizes. (a) MRE of three algorithms and (b) RMSE of three algorithms.

We unify the privacy budget to 2 and take the datasets of 120K, 240K, 360K, 480K, 600K and then calculate the MRE and RMSE in order to study the impact of data sizes on the two evaluation indicators. This experiment results are shown in Fig 7.

In Fig 7, we can see that, in the calculation of these two indicators, the error of Dwork et al.’s scheme is much larger than Kim et al.’s scheme and our scheme, and the error of our scheme is lower than Kim et al.’s scheme in both indicators, indicating that the data usability and data bias of our scheme are better than those of comparison schemes. With increasing data size, both MRE and RMSE of the three schemes become smaller and smaller. Because the two error indicators are inversely related to the 1/2 power of the number of users, which is explained in “the impact of data sizes on actual and estimated values” and will not be repeated here. Table 7 shows the specific data of MRE (Fig 7(a)) for the different data sizes mentioned above.

**Table 7:** MRE under different data sizes.

Data Size	Dwork et al.	Kim et al.	Our solution	Decline rate
120K	11.7679	0.1475	0.0863	41.49%
240K	8.3694	0.1408	0.0610	56.68%
360K	6.8099	0.1232	0.0537	56.41%
480K	5.8002	0.1047	0.0434	58.55%
600K	5.1945	0.0759	0.0383	49.54%

It can be seen from the table that under different data sizes, our scheme has at least 41.49% lower MRE than Kim et al.’s scheme, suggesting that our scheme outperforms the former one by at least 41.49% in terms of usability.

Therefore, from the perspective of the two evaluation indicators, the effectiveness of our scheme is still better than Kim et al.’s scheme under different data sizes.

## Conclusion

The privacy protection of personal health data needs to balance security and usability, and how to improve data usability while protecting user privacy is the key to mining data value. In this paper, we propose a protection scheme for numerical stream data with single attribute based on local differential privacy. Firstly, the device censors the collected stream data to identify a few salient points, and adopts an adaptive Laplace mechanism to add noise to these salient points; secondly, the service provider reconstructs the noisy data in various ways and selects an error minimization scheme. The experimental results show that using our scheme to protect the privacy of one-dimensional attribute stream data of wearable devices can improve the usability of data by at least 41.49% compared to the scheme of Kim et al.

Our proposal is only for one-dimensional attributes, so we will further study the privacy protection of stream data for multi-dimensional attributes in the future.

## Supporting information

S1 Table Dataset summary. (PDF)

S2 Table Errors under different privacy budgets. (PDF)

S3 Table Errors under different data sizes. (PDF)

## References

[1] Liu Q, Li T, Yu Y, Cai Z, Zhou T. Data Security and Privacy Preserving Techniques for Wearable Devices: A Survey. Journal of Computer Research and Development. 2018;55(1):16.

[2] Papageorgiou A, Strigkos M, Politou E, Alepis E, Solanas A, Patsakis C. Security and Privacy Analysis of Mobile Health Applications: The Alarming State of Practice. IEEE Access. 2018;6:9390–9403. doi: 10.1109/ACCESS.2018.2799522

[3] Ye Q, Meng X, Zhu M, Huo Z. Survey on Local Differential Privacy. Journal of Software. 2018;29(7):25.

[4] Erlingsson U, Pihur V, Korolova A. RAPPOR: Randomized Aggregatable Privacy-Preserving Ordinal Response. In: Proceedings of the 2014 ACM SIGSAC Conference on Computer and Communications Security. CCS’14. New York, NY, USA: Association for Computing Machinery; 2014. p. 1054–1067. Available from: 10.1145/2660267.2660348.

[5] Bassily R, Smith A. Local, Private, Efficient Protocols for Succinct Histograms. In: Proceedings of the Forty-Seventh Annual ACM Symposium on Theory of Computing. STOC’15. New York, NY, USA: Association for Computing Machinery; 2015. p. 127–135. Available from: 10.1145/2746539.2746632.

[6] Duchi JC, Jordan MI, Wainwright MJ. Local Privacy and Statistical Minimax Rates. In: 2013 IEEE 54th Annual Symposium on Foundations of Computer Science; 2013. p. 429–438.

[7] Duchi JC, Jordan MI, Wainwright MJ. Privacy Aware Learning. J ACM. 2014;61(6). Available from: 10.1145/2666468.

[8] Nguyên TT, Xiao X, Yang Y, Hui SC, Shin H, Shin J. Collecting and analyzing data from smart device users with local differential privacy. arXiv preprint arXiv:160605053. 2016.

[9] Kellaris G, Papadopoulos S, Xiao X, Papadias D. Differentially Private Event Sequences over Infinite Streams. Proc VLDB Endow. 2014;7(12):1155–1166. Available from: 10.14778/2732977.2732989.

[10] Errounda FZ, Liu Y. Collective location statistics release with local differential privacy. Future Generation Computer Systems. 2021;124:174–186. Available from: https://www.sciencedirect.com/science/article/pii/S0167739X21001709.

[11] Fang X, Zeng Q, Yang G. Local Differential Privacy for Data Streams. In: Yu S, Mueller P, Qian J, editors. Security and Privacy in Digital Economy. Singapore: Springer Singapore; 2020. p. 143–160.

[12] Wang Y, Wu X, Hu D. Using Randomized Response for Differential Privacy Preserving Data Collection. In: EDBT/ICDT Workshops. vol. 1558; 2016. p. 0090–6778.

[13] Kim JW, Jang B, Yoo H. Privacy-preserving aggregation of personal health data streams. PLOS ONE. 2018;13(11):1–15. Available from: 10.1371/journal.pone.0207639. 30496200 PMC6264901

[14] Xu G, Dong W, Xing J, Lei W, Liu J, Gong L, et al. Delay-CJ: A novel cryptojacking covert attack method based on delayed strategy and its detection. Digital Communications and Networks. 2022. Available from: https://www.sciencedirect.com/science/article/pii/S2352864822000864.

[15] Xu G, Bai H, Xing J, Luo T, Xiong NN, Cheng X, et al. SG-PBFT: A secure and highly efficient distributed blockchain PBFT consensus algorithm for intelligent Internet of vehicles. Journal of Parallel and Distributed Computing. 2022;164:1–11. Available from: https://www.sciencedirect.com/science/article/pii/S0743731522000363.

[16] Liu J, Meng X. Survey on Privacy-Preserving Machine Learning. Journal of Computer Research and Development. 2020;057(002):346–362.

[17] Wang X, Zhang Z. Data division scheme based on homomorphic encryption in WSNs for health care. Journal of medical systems. 2015;39(12):1–7. doi: 10.1007/s10916-015-0340-1 26490146

[18] Xu Z, Luo M, Kumar N, Vijayakumar P, Li L. Privacy-Protection Scheme Based on Sanitizable Signature for Smart Mobile Medical Scenarios. Wireless Communications and Mobile Computing. 2020;2020:1–10.

[19] Kong W, Shen J, Vijayakumar P, Cho Y, Chang V. A practical group blind signature scheme for privacy protection in smart grid. Journal of Parallel and Distributed Computing. 2020;136:29–39. Available from: https://www.sciencedirect.com/science/article/pii/S0743731519301285.

[20] Wei F, Vijayakumar P, Kumar N, Zhang R, Cheng Q. Privacy-Preserving Implicit Authentication Protocol Using Cosine Similarity for Internet of Things. IEEE Internet of Things Journal. 2021;8(7):5599–5606. doi: 10.1109/JIOT.2020.3031486

[21] Liu Y, Yu J, Fan J, Vijayakumar P, Chang V. Achieving Privacy-Preserving DSSE for Intelligent IoT Healthcare System. IEEE Transactions on Industrial Informatics. 2022;18(3):2010–2020. doi: 10.1109/TII.2021.3100873

[22] Hua J, Zhu H, Wang F, Liu X, Lu R, Li H, et al. CINEMA: Efficient and privacy-preserving online medical primary diagnosis with skyline query. IEEE Internet of Things Journal. 2018;6(2):1450–1461. doi: 10.1109/JIOT.2018.2834156

[23] Motiian S, Piccirilli M, Adjeroh DA, Doretto G. Unified deep supervised domain adaptation and generalization. In: Proceedings of the IEEE international conference on computer vision; 2017. p. 5715–5725.

[24] Liu F, Li T. A Clustering K -Anonymity Privacy-Preserving Method for Wearable IoT Devices. Security and Communication Networks. 2018;2018:1–8. doi: 10.1155/2018/4945152

[25] Zhou T, Shen J, He D, Vijayakumar P, Kumar N. Human-in-the-Loop-Aided Privacy-Preserving Scheme for Smart Healthcare. IEEE Transactions on Emerging Topics in Computational Intelligence. 2022;6(1):6–15. doi: 10.1109/TETCI.2020.2993841

[26] Xiong X, Liu S, Li D, Cai Z, Niu X. A comprehensive survey on local differential privacy. Security and Communication Networks. 2020;2020.

[27] Yang M, Guo J, Bai L. A Data Privacy-preserving Method for Students’ Physical Health Monitoring by Using Smart Wearable Devices. In: 2020 IEEE Intl Conf on Dependable, Autonomic and Secure Computing, Intl Conf on Pervasive Intelligence and Computing, Intl Conf on Cloud and Big Data Computing, Intl Conf on Cyber Science and Technology Congress (DASC/PiCom/CBDCom/CyberSciTech); 2020. p. 29–34.

[28] Li Z, Wang T, Lopuhaä-Zwakenberg M, Li N, Škoric B. Estimating Numerical Distributions under Local Differential Privacy. In: Proceedings of the 2020 ACM SIGMOD International Conference on Management of Data. SIGMOD’20. New York, NY, USA: Association for Computing Machinery; 2020. p. 621–635. Available from: 10.1145/3318464.3389700.

[29] Wang N, Xiao X, Yang Y, Zhao J, Hui SC, Shin H, et al. Collecting and Analyzing Multidimensional Data with Local Differential Privacy. In: 2019 IEEE 35th International Conference on Data Engineering (ICDE); 2019. p. 638–649.

[30] Guo N. Research on Frequency Statistics Algorithm of Streaming Data Based on Hybrid Differential Privacy [Master Thesis]. Harbin Institute of Technology. CHN; 2019.

[31] Afrose S, Yao DD, Kotevska O. Measurement of Local Differential Privacy Techniques for IoT-based Streaming Data. In: 2021 18th International Conference on Privacy, Security and Trust (PST); 2021. p. 1–10.

[32] Arcolezi HH, Couchot JF, Bouna BA, Xiao X. Improving the Utility of Locally Differentially Private Protocols for Longitudinal and Multidimensional Frequency Estimates. arXiv preprint arXiv:211104636. 2021.

[33] Wang T, Chen JQ, Zhang Z, Su D, Cheng Y, Li Z, et al. Continuous Release of Data Streams under Both Centralized and Local Differential Privacy. In: Proceedings of the 2021 ACM SIGSAC Conference on Computer and Communications Security. CCS’21. New York, NY, USA: Association for Computing Machinery; 2021. p. 1237–1253. Available from: 10.1145/3460120.3484750.

[34] Kim JW, Lim JH, Moon SM, Jang B. Collecting Health Lifelog Data From Smartwatch Users in a Privacy-Preserving Manner. IEEE Transactions on Consumer Electronics. 2019;65(3):369–378. doi: 10.1109/TCE.2019.2924466

[35] Kim JW, Moon SM, Kang Su, Jang B. Effective Privacy-Preserving Collection of Health Data from a User’s Wearable Device. Applied Sciences. 2020;10(18). Available from: https://www.mdpi.com/2076-3417/10/18/6396.

[36] Dwork C, Lei J. Differential Privacy and Robust Statistics. In: Proceedings of the Forty-First Annual ACM Symposium on Theory of Computing. STOC’09. New York, NY, USA: Association for Computing Machinery; 2009. p. 371–380. Available from: 10.1145/1536414.1536466.

[37] Dwork C, McSherry F, Nissim K, Smith A. Calibrating Noise to Sensitivity in Private Data Analysis. In: Halevi S, Rabin T, editors. Theory of Cryptography. Berlin, Heidelberg: Springer Berlin Heidelberg; 2006. p. 265–284.

[38] MathWorks. Interpolation; 2013 [cited 2022 April 30]. Available from: https://www.mathworks.com/content/dam/mathworks/mathworks-dot-com/moler/interp.pdf.

[39] Reiss A, Stricker D. Introducing a New Benchmarked Dataset for Activity Monitoring. In: 2012 16th International Symposium on Wearable Computers; 2012. p. 108–109.

