# Supplementary material for: Local differential privacy protection for wearable device data
Source: PLoS One. 2022 Aug 17;17(8):e0272766. doi: 10.1371/journal.pone.0272766 (PMC9385068; doi:10.1371/journal.pone.0272766)
Supplement: S1 Table — (PDF) [file pone.0272766.s001.pdf]

S1 Table. Dataset summary.

Dataset number	101	102	103	104	105	106	107	108
Number of instances	600	600	600	600	600	600	600	600
Value range	78~120	74~107	68~94	57~121	70~101	60~104	60~99	66~104
