# Supplementary material for: Local differential privacy protection for wearable device data
Source: PLoS One. 2022 Aug 17;17(8):e0272766. doi: 10.1371/journal.pone.0272766 (PMC9385068; doi:10.1371/journal.pone.0272766)
Supplement: S2 Table — (PDF) [file pone.0272766.s002.pdf]

S2 Table. Errors under different privacy budgets.

	MRE			RMSE		
Privacy budget	0.5	1	2	0.5	1	2
Linear	**0.1292**	**0.0662**	**0.0383**	**13.2004**	**6.7997**	**4.5037**
Pchip	0.1383	0.0717	0.0416	14.4537	7.3646	4.8847
Spline	0.1635	0.0799	0.0466	18.0156	8.1994	5.3600
