# Supplementary material for: Local differential privacy protection for wearable device data
Source: PLoS One. 2022 Aug 17;17(8):e0272766. doi: 10.1371/journal.pone.0272766 (PMC9385068; doi:10.1371/journal.pone.0272766)
Supplement: S3 Table — (PDF) [file pone.0272766.s003.pdf]

S3 Table. Errors under different data sizes.

	MRE					RMSE				
Data Size	120K	240K	360K	480K	600K	120K	240K	360K	480K	600K
Linear	**0.0863**	**0.061**	**0.0537**	**0.0434**	**0.0383**	**9.6957**	**6.8377**	**5.5881**	**4.9978**	**4.5037**
Pchip	0.0936	0.065	0.0575	0.0459	0.0416	10.3896	7.2703	5.964	5.3067	4.8847
Spline	0.1033	0.0718	0.0621	0.0511	0.0466	11.2769	8.0054	6.5701	5.8302	5.3600
